# Supplementary material for: Polyketide synthase gene clusters in the ericoid mycorrhizal fungus Oidiodendron maius: genome-wide prediction, expression profiling, and functional characterization of knockout mutants
Source: Front Plant Sci. 2026 Sep 16;17:1924430. doi: 10.3389/fpls.2026.1924430 (PMC13623903; doi:10.3389/fpls.2026.1924430)
Supplement: Supplementary file 1 [file SupplementaryFile1.zip › Supplementary Materials/Supplementary Folder 1.zip!/Oidma1_AssemblyScaffolds_Repeatmasked/knownclusterblast/cluster51/jgi.p_Oidma1_149728_mibig_hits.html]

| MIBiG Protein | Description | MIBiG Cluster | MiBiG Product | % ID | % Coverage | BLAST Score | E-value |
| --- | --- | --- | --- | --- | --- | --- | --- |
| ADI24937.1 | VrtK | BGC0000168 | Viridicatumtoxin\_biosynthetic\_gene\_cluster | 55 | 98.6381322957 | 601 | 7.4e-172 |
| AAC49200.1 | putative\_p450\_monooxygenase | BGC0000152 | Sterigmatocystin\_biosynthetic\_gene\_cluster | 38 | 92.0233463035 | 356 | 5.4e-98 |
| AAS90102.1 | VerB | BGC0000006 | Aflatoxin\_biosynthetic\_gene\_cluster | 36 | 93.7743190661 | 347 | 1.9e-95 |
| AAS90079.1 | VerB | BGC0000010 | Aflatoxin\_biosynthetic\_gene\_cluster | 36 | 93.7743190661 | 347 | 1.9e-95 |
| AAS90033.1 | VerB | BGC0000008 | Aflatoxin\_biosynthetic\_gene\_cluster | 36 | 93.7743190661 | 347 | 1.9e-95 |
| AAM18769.1 | AflL | BGC0000011 | Aflatoxin/sterigmatocystin\_biosynthetic\_gene\_cluster | 37 | 94.1634241245 | 347 | 3.3e-95 |
| AAS90011.1 | VerB | BGC0000007 | Aflatoxin\_biosynthetic\_gene\_cluster | 36 | 93.7743190661 | 346 | 4.3e-95 |
| BAE71326.1 | desaturase | BGC0000004 | Aflatoxin\_biosynthetic\_gene\_cluster | 36 | 93.7743190661 | 341 | 1.4e-93 |
| AAS90059.1 | VerB | BGC0000009 | Aflatoxin\_biosynthetic\_gene\_cluster | 36 | 94.5525291829 | 340 | 4e-93 |
| OAQ83755.1 | isotrichodermin\_C-15\_hydroxylase | BGC0001358 | Leucinostatins\_biosynthetic\_gene\_cluster | 35 | 96.1089494163 | 299 | 7.8e-81 |
| AAS90010.1 | AvnA | BGC0000007 | Aflatoxin\_biosynthetic\_gene\_cluster | 36 | 95.719844358 | 298 | 1.3e-80 |
| BAE71325.1 | cytochrome\_P450\_monooxygenase | BGC0000004 | Aflatoxin\_biosynthetic\_gene\_cluster | 36 | 94.3579766537 | 296 | 6.6e-80 |
| AAS90078.1 | AvnA | BGC0000010 | Aflatoxin\_biosynthetic\_gene\_cluster | 36 | 94.3579766537 | 296 | 6.6e-80 |
| AAS90101.1 | AvnA | BGC0000006 | Aflatoxin\_biosynthetic\_gene\_cluster | 36 | 94.3579766537 | 295 | 8.7e-80 |
| AAS90032.1 | AvnA | BGC0000008 | Aflatoxin\_biosynthetic\_gene\_cluster | 36 | 94.3579766537 | 295 | 1.5e-79 |
| ACH72905.1 | AflG | BGC0000011 | Aflatoxin/sterigmatocystin\_biosynthetic\_gene\_cluster | 35 | 95.1361867704 | 291 | 1.6e-78 |
| AAS90058.1 | AvnA | BGC0000009 | Aflatoxin\_biosynthetic\_gene\_cluster | 36 | 96.4980544747 | 291 | 2.1e-78 |
| ACD39773.1 | cytochrome\_P450 | BGC0000134 | Radicicol\_biosynthetic\_gene\_cluster | 34 | 95.1361867704 | 287 | 3.1e-77 |
| AAK33080.1 | putative\_trichothecene\_C-15\_hydroxylase | BGC0001277 | Nivalenol\_/\_deoxynivalenol\_/\_3-acetyldeoxynivalenol\_/ | 37 | 94.1634241245 | 286 | 6.9e-77 |
| AAK53587.1 | isotrichodermin\_C-15\_hydroxylase | BGC0000931 | Trichothecene\_biosynthetic\_gene\_cluster | 37 | 92.4124513619 | 284 | 2.6e-76 |
| AAK33070.1 | trichothecene\_C-15\_hydroxylase | BGC0001278 | Nivalenol\_/\_deoxynivalenol\_/\_3-acetyldeoxynivalenol\_/ | 37 | 92.2178988327 | 282 | 7.6e-76 |
| BAD83682.1 | cytochrome\_P-450 | BGC0000012 | Alternapyrone\_biosynthetic\_gene\_cluster | 33 | 94.3579766537 | 282 | 9.9e-76 |
| BAE56591.1 |  | BGC0001123 | Aspirochlorine\_biosynthetic\_gene\_cluster | 35 | 92.2178988327 | 280 | 2.9e-75 |
| AAC49196.1 | putative\_p450\_monooxygenase | BGC0000152 | Sterigmatocystin\_biosynthetic\_gene\_cluster | 35 | 91.2451361868 | 279 | 8.4e-75 |
| AAK53580.1 | isotrichodermin\_C-15\_hydroxylase | BGC0000930 | Trichothecene\_biosynthetic\_gene\_cluster | 37 | 91.8287937743 | 277 | 4.2e-74 |
| ACD39760.1 | cytochrome\_P450 | BGC0000077 | Hypothemycin\_biosynthetic\_gene\_cluster | 34 | 90.8560311284 | 272 | 1e-72 |
| ACD39751.1 | cytochrome\_P450 | BGC0000076 | Hypothemycin\_biosynthetic\_gene\_cluster | 34 | 90.8560311284 | 272 | 1e-72 |
| Bcin12g06370.1 |  | BGC0000631 | Botrydial\_biosynthetic\_gene\_cluster | 35 | 92.9961089494 | 259 | 1.2e-68 |
| BAK26559.1 | cytochrome\_P450 | BGC0000977 | Cyclopiazonic\_acid\_biosynthetic\_gene\_cluster | 32 | 89.6887159533 | 250 | 4.2e-66 |
| BAD29972.1 | P450\_monooxygenase\_1 | BGC0000676 | Aphidicolin\_biosynthetic\_gene\_cluster | 33 | 94.9416342412 | 247 | 2.7e-65 |
| XP\_002384857.1 | cytochrome\_P450,\_putative | BGC0001234\_c2 | 4,4'-piperazine-2,5-diyldimethyl-bis-phenol\_biosynthetic\_gene | 32 | 93.3852140078 | 234 | 2.4e-61 |
| BAO10618.1 | cytochrome\_P450\_monooxygenase | BGC0001262 | AK-toxin\_biosynthetic\_gene\_cluster | 28 | 88.1322957198 | 180 | 6.9e-45 |
| AAZ95016.1 | cytochrome\_P450\_monooxygenase | BGC0000048 | Dothistromin\_biosynthetic\_gene\_cluster | 28 | 94.5525291829 | 156 | 1.1e-37 |
| ADI24958.2 | GsfF | BGC0000070 | Griseofulvin\_biosynthetic\_gene\_cluster | 23 | 94.9416342412 | 108 | 2e-23 |
| ABI22130.1 | cytochrome\_P450\_hydroxylase | BGC0000422 | Saframycin\_A\_biosynthetic\_gene\_cluster | 24 | 39.1050583658 | 53 | 9.8e-07 |
